# Interpretable spatial identity neural network-based epidemic prediction

**DOI:** 10.1038/s41598-023-45177-1

**Published:** 2023-10-24

**Authors:** Lanjun Luo, Boxiao Li, Xueyan Wang, Lei Cui, Gang Liu

**Affiliations:** 1 School of Management, North Sichuan Medical College, Nanchong, China; 2 Information Centre, Affiliated Hospital of North Sichuan Medical College, Nanchong, China; 3 Graduate School of Information Sciences, Tohoku University, Sendai, Japan; 4 School of Artificial Intelligence and Automation, Huazhong University of Science and Technology, Wuhan, China; 5 School of Management, Huazhong University of Science and Technology, Wuhan, China

**Keywords:** Applied mathematics, Computer science

## Abstract

Epidemic spatial–temporal risk analysis, e.g., infectious number forecasting, is a mainstream task in the multivariate time series research field, which plays a crucial role in the public health management process. With the rise of deep learning methods, many studies have focused on the epidemic prediction problem. However, recent primary prediction techniques face two challenges: the overcomplicated model and unsatisfactory interpretability. Therefore, this paper proposes an Interpretable Spatial IDentity (ISID) neural network to predict infectious numbers at the regional weekly level, which employs a light model structure and provides post-hoc explanations. First, this paper streamlines the classical spatio-temporal identity model (STID) and retains the optional spatial identity matrix for learning the contagion relationship between regions. Second, the well-known SHapley Additive explanations (SHAP) method was adopted to interpret how the ISID model predicts with multivariate sliding-window time series input data. The prediction accuracy of ISID is compared with several models in the experimental study, and the results show that the proposed ISID model achieves satisfactory epidemic prediction performance. Furthermore, the SHAP result demonstrates that the ISID pays particular attention to the most proximate and remote data in the input sequence (typically 20 steps long) while paying little attention to the intermediate steps. This study contributes to reliable and interpretable epidemic prediction through a more coherent approach for public health experts.

## Introduction

Infectious disease epidemics are highly susceptible to significant adverse effects on social function due to the contagion characteristics involving a large number of people, wide geographic area, and high speed. For example, within the first two years of the new pandemic, COVID-19 was identified as the third leading cause of death in the United States, after heart disease and cancer^1^. Therefore, predicting the number of infections at a finer spatial and temporal scale will facilitate timely intervention and resource allocation, essential for developing prospective epidemic prevention policies.

Currently, with the benefit of publicly available data from various countries' CDCs, researchers can obtain regional-weekly level data on the infection numbers, forming a high-quality multivariate time series (MTS) dataset. High-quality MTS data provides the possibility for accurate epidemic prediction; thus, many studies have focused on this problem. Recently, with the rise of deep learning methods, various deep learning-based epidemic prediction models have been proposed. The most representative is the graph representation learning method for epidemic prediction^2^, which is regarded as a better way to handle epidemic prediction tasks than traditional recurrent neural network (RNN) or convolutional neural network (CNN) ideas that mainly deal with conventional raster data. However, although it has been demonstrated that graph representation learning methods can achieve promising epidemic prediction results, there are still two shortcomings from the perspective of serving public health management: the overcomplicated model and unsatisfactory interpretability.

The current mainstream epidemic prediction models mainly employ the combination of graph representation learning layers with other neural network layers. The complexity of the models is highly noticeable: progressively deeper layers of neural networks, the mixture of multiple neuron structures, etc. The most typical, the latest Cola-GNN proposed by Deng et al.^3^, combines the graph information transfer layer, 1D-CNN layer, and RNN layer. However, the endless refinements of neural network models have often resulted in incremental improvements of predictions, but the large and overcomplicated computational models themselves may become increasingly difficult to understand for healthcare and public health experts.

Thus, the challenge of prediction model interpretability arises. Prediction models are required to maintain reliable predictive performance and understandability for users, especially concerning the inherent computational rules and decision mechanisms. However, over-sophisticated deep learning models usually have a vast number of parameters and lack interpretable rules; hence are commonly criticized as "black boxes" that are difficult to understand. This means that many epidemic prediction models focus on improving accuracy and can only present the results of epidemic risk predictions without providing deeper insight into the underlying causes. Simply knowing the predicted value without understanding why and how an increase in infections occurs and which factors are most critical could make it difficult to prospectively develop public health strategies and manage epidemic outbreak risk.

Therefore, to deal with the challenges of overcomplicated models and unsatisfactory interpretability, this study proposed an intuitive neural network model named Interpretable Spatial IDentity (ISID) to perform an understandable prediction of infectious disease numbers at the regional weekly level. First, this study modified the well-known Spatio-Temporal IDentity (STID)^4^ model to perform the epidemic prediction. The temporal identity module in the classical STID model is removed, and the spatial trainable matrix is retained for capturing the epidemic contagion pattern. Second, influenza data from the United States and Japan were used to compare the ISID model with other complex deep learning models, demonstrating that simple models that are sufficiently refined can also achieve decent performance. Third, the SHapley Additive exPlanations (SHAP) post-hoc interpretable method explains how the ISID model uses multivariate sliding window input data for prediction, analyzing which time step features are crucial to explain the model's decision basis.

The main contribution of this paper can be summarized as threefold:

- A novel interpretable epidemic prediction model is designed, which is more straightforward than traditional complex models while achieving reliable performance.
- Considering the contagion of epidemics in space–time, a learnable spatial matrix is applied to explore the potential spatio-temporal correlations between regions.
- For the spatio-temporal epidemic prediction task with three-dimensional prediction inputs, the SHAP method is used to innovatively explain the effect of different step features on model prediction.

The rest of the paper is organized as follows: Section "Related works" reviews related works. Section "Methodology" presents the ISID model framework of this study. The experimental results are shown in "Experimental study". Finally, the work is summarized, and future research is discussed in "Conclusion".

## Related works

### Epidemic prediction

One essential purpose of epidemic prediction is to forecast the number of infected cases in selected spaces at particular periods based on historical data^5^. Due to the vital relevance and universality of such a task, many studies are already focusing on epidemic prediction, which can be divided methodologically into two main categories: traditional statistical models and deep learning models.

The most classical idea for predicting the number of infections in the context of an epidemic is the Susceptible-Infective-Recovered (SIR) model and its variants^6–8^. These models mainly use a combination of priori parameters setting and differential equation modeling, using historical data for parameter estimation and predicting the future trend of the infection numbers. In addition, many studies analyzed from a time series perspective have also extensively used classical statistical modeling methods, such as the well-known AutoRegressive Integrated Moving Average (ARIMA)^9,10^, Logistic regression^11^, etc. However, such ideas are highly dependent on constructing parametric models and selecting parameters, which are usually ineffective in handling complex nonlinear relationships and challenging to accommodate multi-spatial-multi-timestep spatio-temporal epidemic prediction tasks considering the epidemic transmission characteristics.

In recent years, due to the outstanding achievements of deep learning in multidisciplinary fields, many studies based on this method for epidemic prediction have achieved better results than traditional statistical models. Typically, Tsan et al.^12^ adopted the Long Short-Term Memory (LSTM) neural networks to predict influenza-like illness and respiratory disease, and LSTM is superior to ARIMA. Alkouz et al.^13^ proposed a Bidirectional Encoder Representation from Transformers (BERT) based influenza detection model, outperforming traditional methods. Yang et al.^14^ also used the LSTM method to predict epidemics through multiple open data sources. Jung et al.^15^ proposed a self-attention (SA) based model for regional influenza prediction, which combines LSTM and SA structures and outperforms other comparative methods in terms of effectiveness. However, the essential characteristic of infectious diseases, i.e., contagiousness, has received scant attention in these studies. Related methods lack modeling and learning about the mobility of spatial transmission risk at different times, and less discuss epidemics’ time-varying nature and association.

Recently, deep learning methods based on graph representation learning have made achievements in dealing with spatial correlations. This approach copes well with irregular, non-Euclidean graph-structured data and exceeds the performance of standard deep learning methods in many spatio-temporal prediction tasks. For example, the Spatio-Temporal Graph Convolutional Network (STGCN) proposed by Yu et al.^16^ has become a benchmark for traffic flow prediction and is also used for comparison in many epidemic spatio-temporal prediction tasks^3,5^. Although the graph representation learning approach seems to be state-of-the-art, some scholars have found that it still has shortcomings.

The most critical challenge is that deep learning methods increasingly rely on complex neural network structures, usually combining modules such as graph convolutional neural networks, LSTM, and self-attention, making the internal structure of the models more complicated, which may bring limited predictive performance improvements. For example, Elsayed et al.^17^ found that the classical Gradient Boosting Regression Tree (GBRT) model performs significantly better than complex deep learning models on multiple datasets, and deep learning models are probably overly complex. Shao et al.^4^ proposed the Spatio-Temporal Identity (STID) model, which generates trainable matrices for representing spatial spreading effects and temporal features in spatio-temporal prediction tasks, exhibiting better applicability than graph representation learning models. These findings suggest that perhaps a simpler model would be a better option for spatiotemporal forecasting.

Therefore, in this study, the STID model is modified to a simpler version with a spatial identify matrix to learn the contagion pattern of epidemics in different spaces. In the following, this paper reviews another problem faced by deep learning prediction methods: the lack of interpretability.

### Interpretable machine learning

Interpretability refers to the degree to which a model’s prediction and decision process can be understood, and higher interpretability means that the model is more credible, reliable, and transparent to the user^25^. Although machine learning and deep learning models have efficient nonlinear fitting capability, they are usually considered black boxes and lack interpretability due to their huge number of parameters and complex structure, making them difficult to understand by users as classical regression models. Therefore, in order to exploit the efficient performance of machine learning and to improve its comprehensibility, many works have started to investigate interpretable machine learning in recent years^18^. These studies intend to anatomize the correlation and causality between input–output features learned by the model to the user, thus analyzing the mechanism of model decisions. Specifically, the current mainstream explainable machine learning techniques can be divided into intrinsically explainable methods and post-hoc explainable methods.

The intrinsically interpretable approach is a model-related explainability implementation method, which implies that the structure of the model itself is easily understandable and the decision process is straightforward. The most usual intrinsically interpretable methods are classical models such as regression and decision trees^19^. These models have more stringent assumptions and constraints, such as linear additivity of regression models, conditional independence of Naive Bayes, branching rules of decision trees, etc.^20–22^. Although the above models can characterize the weights, positive and negative correlations, and contributions of different influencing factors to the dependent variable, the drawback is the limited ability to fit the nonlinear relationships and the poor prediction accuracy. Another representative of the intrinsically interpretable approach is the attention mechanism commonly used in neural network models, which can analyze the computational rules of the model concerning the inputs through the attention-weighted matrix^23,24^. However, the limitation of the attention mechanism is that it is challenging to build a neural network model entirely on this structure, and in complex deep learning models with deeper layers, the attention mechanism can only ensure partial interpretability but not the interpretability of the whole model.

Post-hoc interpretable methods are the most mainstream and widely used ideas, usually not dependent on specific model structures and assumptions. This approach requires the model to undergo a training-fitting process and produce prediction outputs before the decision process can be analyzed, and is therefore referred to as “Post-hoc” interpretability^25^. The post-hoc interpretable methods can be divided into global and local interpretations, where global interpretation refers to the model’s overall behavior and decision rules over the entire dataset. In contrast, local interpretation refers to the decision basis of the model for single or partial samples. The global interpretation method mainly includes permutation Feature Importance (FI) analysis^26,27^, Partial Dependence Plot (PDP)^28^, and Accumulated Local Effect (ALE)^29^, etc. The FI method intends to measure the change of input factors on the model prediction performance and then measure the importance of different factors on the dependent variable and identify the most crucial terms. The PDP and ALE methods calculate the average effect of different factors on the dependent variable over the entire dataset by replacing the factor values, presented as a binary or multivariate nonlinear relationship between particular influencing factors and the dependent variable.

Although global post-hoc interpretable methods can provide richer explanatory results than intrinsic interpretable approaches, these methods still lack analysis of local samples. The typical local post-hoc interpretable method is the Shapley Additive xplanation (SHAP)^30^. The SHAP method can measure the net contribution of each input factor to the predicted value for the selected sample based on game-theoretic ideas, which in turn explains the predictive decision composition for that instance. Based on this idea, SHAP can be further extended to results such as feature importance measured by multiple instances. Due to its flexible thinking and extensive explanation results, SHAP has been applied in several fields, such as length of hospitalization^31^, environmental quality analysis^32^, construction research^33^, etc.

As can be seen, the above-mentioned related research does not focus on the issue of epidemic prediction analysis with multivariate time series characteristics, nor do they explore this issue using interpretable machine learning methods. Identifying critical influencing factors and analyzing the epidemic contagion process is difficult, resulting in insufficient support for practical risk prevention and preparation. Therefore, this study combines SHAP and prediction models for interpretable spatio-temporal prediction of epidemics.

## Methodology

### Epidemic prediction task

The epidemic prediction task can be considered a spatio-temporal forecasting problem with a resolution of week-region. The general idea is to use the observed historical weekly records of infection numbers in $n$ regions to predict future infections. The historical data can be represented as $H=[{h}_{1},{h}_{2},\dots ,{h}_{t}]$, where $H$ denotes a dataset containing $t$ weeks’ records and each region having one epidemic statistic result in each week, ${h}_{t}=[{x}_{1}^{t},{x}_{2}^{t}\dots {x}_{n}^{t}]\in {\mathbb{R}}^{n}$ denotes the data of $n$ regions on the $t$-th week, and ${x}_{n}^{t}$ denotes the corresponding infection numbers of the $n$-th region on week $t$. For the prediction task, the main idea is to predict the future infection number on week $\alpha$ using the past $\Delta$ weeks’ lookback window data for the $n$ regions, which can be represented as Eq. (1):

1 $${H}_{t+\alpha }=f({H}_{t-\Delta +1},{H}_{t-\Delta +2},\dots ,{H}_{t})$$

where $\alpha$ denotes the prediction ahead timestep, defined as the future infection number on week $\alpha$, and $f$ denotes the epidemic prediction mapping. ${H}_{t-\Delta +1},{H}_{t-\Delta +2},\dots ,{H}_{t}$ denotes the past $\Delta$-length records, which can also be represented as ${H}_{t-\Delta +1:t}\in {\mathbb{R}}^{\Delta \times n}$, $\Delta$ denotes the lookback sliding window length.

### ISID model structure

**Figure 1:**
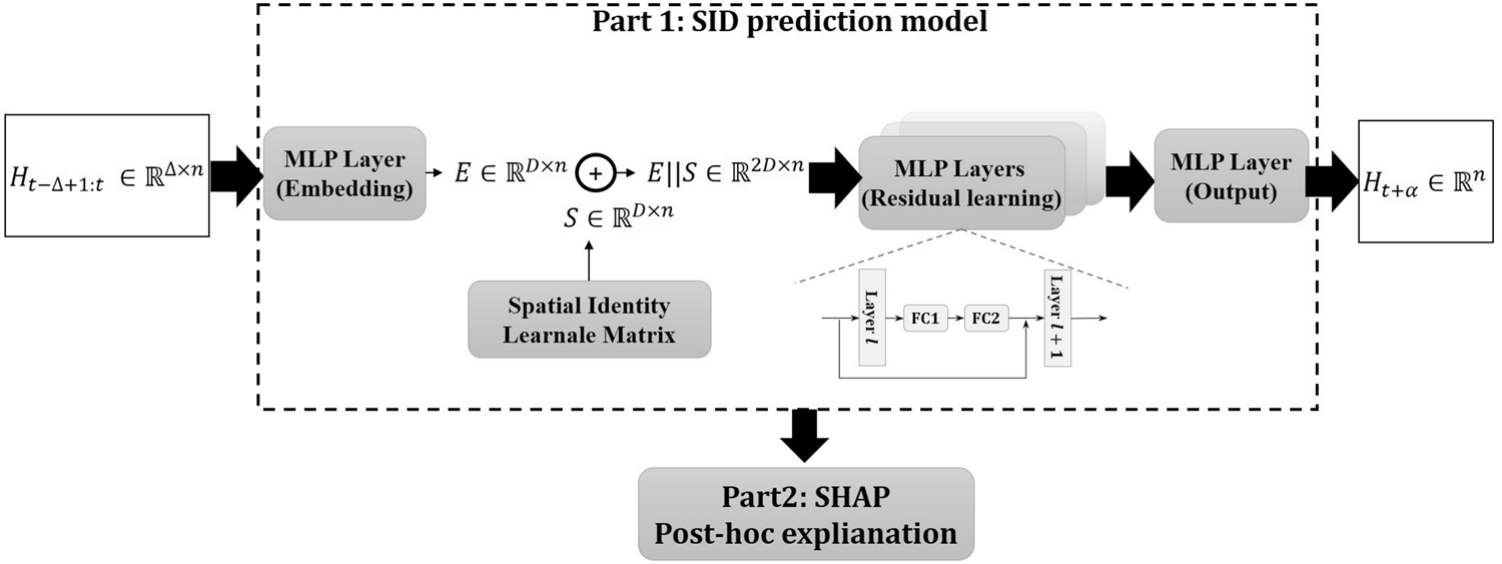
The overall structure of the ISID model.

This study utilizes the ISID model to perform the regional weekly infection number prediction task. The model is divided into two main parts. First, the main structure of the model is streamlined on the classical STID model, using a learnable spatial embedding matrix to learn the cross-regional contagion pattern. Second, the well-known SHapley additive interpretation (SHAP) method is used to explain how the ISID model uses multivariate sliding window input data for prediction. The overall structure of the ISID model is shown in Fig. 1.

Along the lines of STID, this study hopes to build prediction models using the most straightforward fully-connected layers that public health experts can understand without long-term experience in complex deep learning model building. As shown in Fig. 1, three MultiLayer Perceptron (MLP) fully connected layers are responsible for input embedding, residual learning, and output regression, respectively. For the embedding MLP layer, denote ${H}_{t-\Delta +1:t}\in {\mathbb{R}}^{\Delta \times n}$ as a set of input, the calculation inside it can be represented as Eq. (2):

2 $$E={MLP}_{Embedding}({H}_{t-\Delta +1:t})\in {\mathbb{R}}^{D\times n}$$

where ${MLP}_{Embedding}$ denotes the first MLP layer. $E$ denotes the embedded input tensor, and $D$ denotes the embedding dimension. It can be seen that the input timestep length is changed from $\Delta$ to $D$. The purpose is to perform time-dimensional feature learning through the embedding layer.

The second and most crucial component is to generate a learnable spatial identity matrix. Unlike the idea of general graph neural networks that use graph convolution to obtain information transfer or epidemic contagion between different spaces, the idea adopted in the ISID model is to generate a trainable matrix $S\in {\mathbb{R}}^{D\times n}$ and iteratively learn for this spatial matrix in the backpropagation optimization process, and finally treat the matrix as an expression of the different spatial proximity relationships. The spatial identity matrix is denoted as $S\in {\mathbb{R}}^{D\times n}$, means that for each of the $n$ regions, an embedding representation of length $D$ is generated, representing the coordinates in the high-dimensional space that are learned and characterized. Immediately following, the ISID model utilizes the idea of concat to combine the embedded input $E$ with the generated spatial identity representation $S$, as shown in Eq. (3):

3 $$\mathrm{concat}(E,S)=E||S\in {\mathbb{R}}^{2D\times n}$$

Subsequently, the residual learning MLP layers are used as the primary ISID learning structure. It consists of multiple MLP layers, and the total number of MLP is denoted by $L$. Each MLP layer contains two fully connected (FC) modules, which are calculated as Eq. (4):

4 $${(E||S)}^{l+1}={FC}_{2}^{l}\left(\sigma ({FC}_{1}^{l}({(E||S)}^{l}))\right)+{(E||S)}^{l}\in {\mathbb{R}}^{2D\times n}$$

where ${(E||S)}^{l}$ denotes the input of $l$-th residual learning MLP layer, ${FC}_{1}^{l}$ denotes the first FC module of the $l$-th MLP, ${FC}_{2}^{l}$ denotes the second FC module, and $\sigma$ represents the composite of activation function and dropout mechanism. The purpose of designing such a residual structure is to avoid overfitting while deepening the model layers and learning more data patterns simultaneously. It is worth noting that during this process, the embedding dimension of the input remains constant at $2D$.

Finally, the ISID gives specific predictions ${H}_{t+\alpha }\in {\mathbb{R}}^{n}$ through the MLP output layer, which can be represented as Eq. (5):

5 $${H}_{t+\alpha }={MLP}_{output}({MLP}_{Residual}(E||S))\in {\mathbb{R}}^{n}$$

where ${MLP}_{Residual}$ denotes the L-layer residual learning MLP, ${MLP}_{output}$ denotes the single-layer output part. ${H}_{t+\alpha }$ denotes the entire prediction result. The pseudocode of the ISID model is described in Algorithm 1.


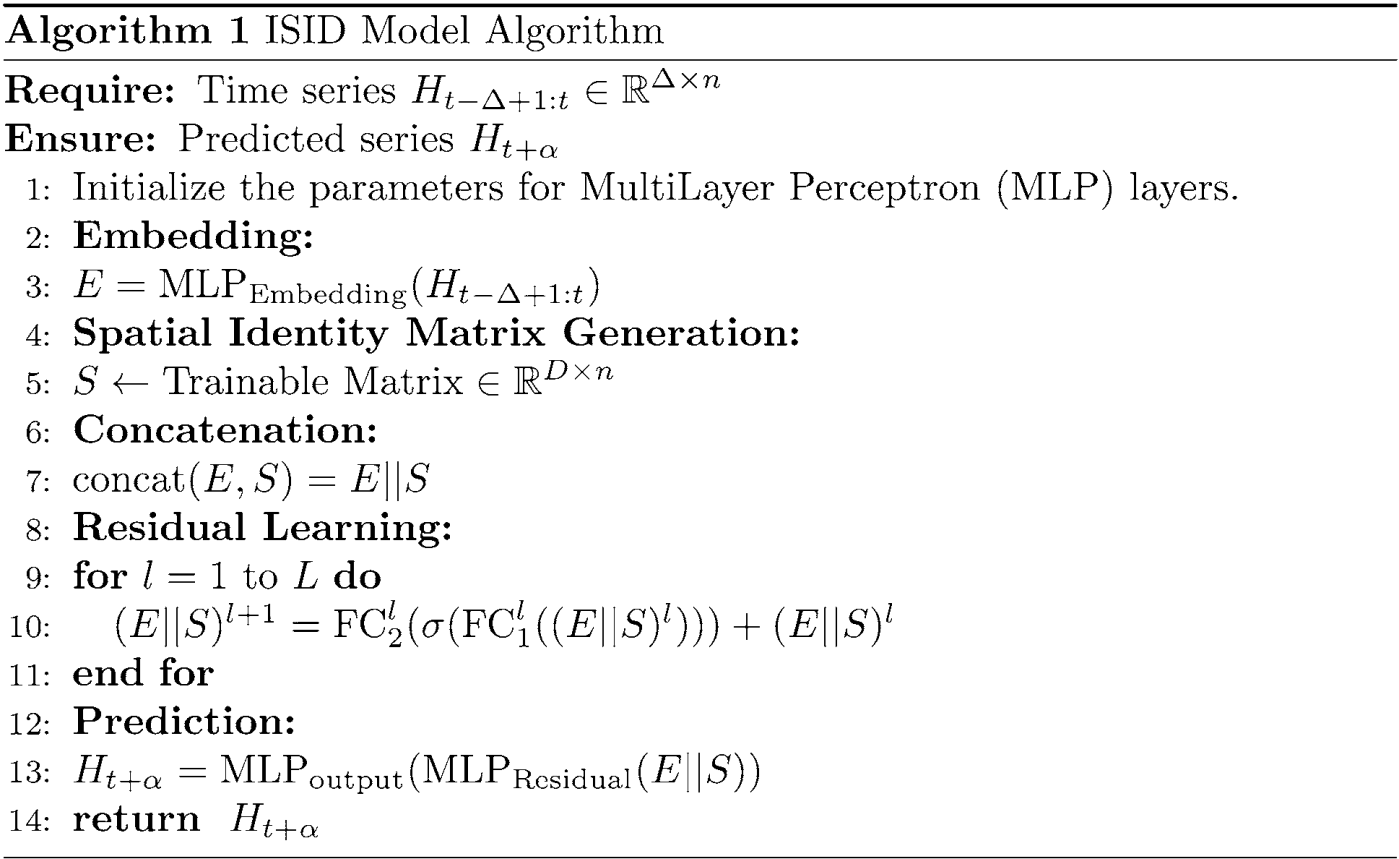


### SHAP explanation

In the second stage of ISID, namely the post-hoc interpretable part, after obtaining the specific prediction results, this study uses the SHAP method to analyze the model’s decision process. The innovative extension of SHAP to multivariate time series input data in this study is because the epidemic prediction task does not use traditional tabular data, but multiple regions-multiple historical time steps of input. This means that the initial format of each input is always two-dimensional, i.e., ${H}_{t-\Delta +1:t}\in {\mathbb{R}}^{\Delta \times n}$. As a result, the interpretation results will be presented in a pattern similar to the concentrated image areas, with inputs that have a more positive impact on the model predictions being marked in a redder color, and inputs that have a more negative impact in a bluer color, with a gray color between red and blue meaning that the input has limited impact on the model. The SHAP calculation details can be found in Appendix A1.

## Experimental study

### Dataset and measurement

For the experimental part, this study adopted two well-known epidemic open datasets for training, validation, and testing: US-Regions and Japan-Prefectures seasonal influenza datasets. US-Regions consists of weekly influenza infection numbers recorded by the United States Department of Health and Human Services from 2002 to 2017. The Japan-Prefectures dataset from the Japan Infectious Diseases Weekly Report contains weekly infection numbers for 47 prefectures from August 2012 to March 2019. The descriptive statistics of two datasets are shown in Table 1, where Regions represent the number of spatial areas recorded, Timeslots represents the total number of sequential records, Mean and Std represent the mean and standard deviation of infection numbers, and the spatio-temporal resolution of two datasets is in the last two columns, both recorded once a week.

**Table 1:** Statistics of datasets.

Datasets	Regions	Timeslots	Infections mean	Infections std	Spatial resolution	Temporal resolution
US-Regions	10	785	1009	1351	Regions	Weekly
Japan-Prefectures	47	348	655	1711	Prefectures	Weekly

The assessment of the predictive effectiveness of the model on different datasets is in line with the epidemic prediction research tradition, and this study uses Root Mean Square Error (RMSE) and Pearson Correlation (PCC), Mean Absolute Scaled Error (MASE), and Mean Absolute Error (MAE) as the outcome measures. For the RMSE, MASE, and MAE values, smaller is better, while for PCC values, larger is better, which can be calculated as Eqs. (6)–(9). Further, considering the characteristics of multivariate time series prediction task, this study also adopts the Diebold Mariano (DM) test^34^ to pairwise analyse the efficiency among algorithms, and the calculation can be found in Appendix A2.

6 $$\mathrm{RMSE}=\sqrt{\frac{1}{n}\sum_{i=1}^{n}{({\widehat{y}}_{i}-{y}_{i})}^{2}},$$

7 $$\mathrm{PCC}=\frac{{\sum }_{i=1}^{n}({\widehat{y}}_{i}-\overline{\widehat{y} })({y}_{i}-\overline{y })}{\sqrt{\sum_{i=1}^{n}{({\widehat{y}}_{i}-\overline{\widehat{y} })}^{2}}\sqrt{\sum_{i=1}^{n}{({y}_{i}-\overline{y })}^{2}}}$$

8 $$\mathrm{MAE}=\frac{1}{n}{\sum }_{i=1}^{n}\left|{\widehat{y}}_{i}-{y}_{i}\right|$$

9 $$\mathrm{MASE}=\frac{\mathrm{MAE}}{\frac{1}{T-1}{\sum }_{t=2}^{T}\left|{y}_{t}-{y}_{t-1}\right|}$$

where ${y}_{i}$ denotes the $i$-th ground truth in the test set with total $n$ samples, ${\widehat{y}}_{i}$ denotes the corresponding prediction from a specific model, $\overline{\widehat{y} }$ and $\overline{y }$ represent the mean of the test set and predicted values. For MASE, the numerator denotes the MAE on the test set, and the denominator is calculated on the training set, ${y}_{t}$ denotes the $t$-th ground truth in the training set, and $T$ is the total training sample size.

### Hyperparameters and comparison models

This study implements the ISID model using the Python deep learning framework PyTorch^35^. Follow the related latest comparative experimental analysis approach from Cola-GNN^36^, several models were used for comparison: autoregressive (AR), autoregressive moving average (ARMA), vector auto regression (VAR), global auto regression (GAR), recurrent neural network (RNN), self-attention with RNN (ATTRNN)^37^, diffusion convolution recurrent neural network (DCRNN)^38^, Long- and Short-term time-series network (LSTNet)^39^, STGCN^16^, and Cola-GNN^36^.

The comparison methods can be divided into traditional models and deep models. The AR, ARMA, VAR, and GAR are four classical autoregressive approaches and variants, whereas the RNN model consists of a simple RNN layer and an output layer without a complex structure. Therefore, these five models are considered as the traditional models. Among the other five models, the ATTRNN combines deep attention fusion with RNN, and the DCRNN adopts bidirectional random walks on the graph representation and encoder-decoder architecture. STGCN is one of the best benchmark graph neural networks-based models in the spatio-temporal prediction research field, and Cola-GNN is the latest epidemic prediction model available. Due to the relatively elaborate multi-layered structures, these five models are considered deep models.

For the ISID model, a lighter variant named ISID-w/o without the spatial identity learnable matrix is also used in the experiments. The embedding dimension *D* for both ISID and ISID-w/o in Eqs. (2)–(4) is set to 32, and the number of residual learning MLP layers *L* is set to 2. The past lookback window size $\Delta$ is set to 20, and the prediction ahead timestep $\alpha$ is set to 3, 5, and 10. The batch size is 32, and the number of epochs is 50. The optimizer adopted in this study is the Adam; the initial learning rate is set to 0.001, the learning rate weight decays is set to 0.0005, and the loss function is the L1 loss. The project of this study is available at https://github.com/minasora/ISID.

### Comparison of model performances

In order to robustly test the predictive effectiveness of the models and avoid problems such as possible overfitting due to fixed training, validation, and test sets, this study adopts the Time Series Cross-Validation (TSCV) method^40^ to report the performances. The details of TSCV can be found in Appendix A3. The datasets are sequentially divided into training and validation sets for TSCV (80%) and test sets (20%) for model prediction evaluation. The performances of all models on the two datasets are shown in Table 2. The experiment is implemented using PyTorch 1.11.0 with CUDA 11.3 with an Nvidia RTX 4090 GPU.

**Table 2:** Performance of different prediction models on two datasets ($\alpha =3, 5, 10$).

$\mathrm{\alpha }$	Models	Japan-Prefectures					US-Regions				
		Time↓	MAE↓	RMSE↓	PCC↑	MASE↓	Time↓	MAE↓	RMSE↓	PCC↑	MASE↓
3	AR	–	901.092	2301.890	0.408	17,307.353	–	687.998	1202.913	0.726	11,331.757
	ARMA	–	893.264	2307.123	0.407	17,157.001	–	537.870	967.471	0.815	8859.046
	VAR	–	907.815	2134.757	0.528	17,436.482	–	668.485	1068.316	0.752	11,010.370
	GAR	–	849.242	2213.338	0.480	16,311.478	–	551.869	990.890	0.846	9089.628
	RNN	–	781.750	2132.552	0.530	15,015.145	–	441.041	865.624	0.869	7264.217
	ATTRNN	40.355	933.302	2411.137	0.500	17,926.013	36.136	1004.317	1645.351	0.480	16,541.732
	DCRNN	200.051	895.577	2335.206	0.402	17,201.437	272.441	766.220	1323.303	0.751	12,620.119
	LSTNet	8.701	662.767	1751.503	0.724	12,729.834	9.352	*427.473*	**834.037**	*0.871*	*7040.749*
	STGCN	17.288	723.386	1835.250	0.727	13,894.146	16.296	717.316	1282.217	0.720	11,814.639
	Cola-GNN	36.980	626.126	*1640.435*	**0.768**	12,026.058	13.835	555.772	1061.352	0.769	9153.913
	ISID	*8.104*	**577.497**	**1622.780**	*0.765*	**11,092.046**	**5.987**	486.672	947.311	0.862	8015.791
	ISID-w/o	**7.569**	*579.743*	1653.814	0.758	*11,135.177*	*6.636*	**416.454**	*840.721*	**0.887**	**6859.255**
5	AR	–	1016.062	2511.999	0.230	19,803.269	–	772.012	1290.221	0.696	12,696.277
	ARMA	–	1006.623	2498.430	0.244	19,619.301	–	745.912	1264.931	0.698	12,267.039
	VAR	–	1086.820	2489.193	0.241	21,182.367	–	740.790	1189.132	0.693	12,182.811
	GAR	–	1046.382	2527.615	0.205	20,394.219	–	766.383	1332.400	0.729	12,603.710
	RNN	–	935.576	2460.424	0.285	18,234.579	–	*613.253*	*1138.828*	0.770	*10,085.375*
	ATTRNN	39.867	944.414	2439.369	0.499	18,406.849	35.844	1111.614	1783.450	0.406	18,281.268
	DCRNN	159.379	989.724	2543.968	0.179	19,289.936	266.518	978.696	1542.252	0.702	16,095.335
	LSTNet	4.704	946.996	2384.207	0.323	18,457.165	8.627	654.376	1189.986	0.716	10,761.663
	STGCN	9.176	778.483	*1916.113*	*0.650*	15,172.816	16.765	975.935	1622.866	0.608	16,049.926
	Cola-GNN	17.099	791.589	1956.292	**0.667**	15,428.257	18.298	639.610	1185.155	*0.800*	10,518.829
	ISID	**4.014**	**721.849**	**1893.874**	0.639	**14,068.998**	*7.330*	672.278	1220.182	0.781	11,056.081
	ISID-w/o	*4.083*	*738.649*	1954.047	0.632	*14,396.427*	**7.166**	**559.990**	**1063.651**	**0.821**	**9209.433**
10	AR	–	1046.727	2541.373	0.307	21,559.834	–	1122.422	1760.956	0.446	18,319.391
	ARMA	–	1030.423	2532.513	0.329	21,224.012	–	1132.658	1781.562	0.441	18,486.455
	VAR	–	1055.402	2506.778	0.298	21,738.525	–	1012.296	1582.399	0.435	16,521.980
	GAR	–	1125.465	2648.507	0.151	23,181.629	–	1062.144	1711.561	0.505	17,335.567
	RNN	–	947.394	2419.028	0.306	19,513.847	–	905.899	1552.777	0.601	14,785.453
	ATTRNN	21.100	984.865	2476.640	0.271	20,285.636	35.535	1092.366	1781.418	0.428	17,828.839
	DCRNN	122.919	1005.184	2554.305	0.352	20,704.160	256.761	1042.101	1652.853	0.669	17,008.447
	LSTNet	*4.573*	1068.836	2559.348	0.186	22,015.237	7.492	**777.090**	**1355.054**	*0.677*	**12,683.112**
	STGCN	9.024	869.911	2239.212	**0.572**	17,917.891	14.377	1033.251	1622.881	0.568	16,864.000
	Cola-GNN	17.097	882.353	*2149.467*	*0.563*	18,174.159	16.470	889.286	*1448.841*	**0.748**	14,514.302
	ISID	**4.137**	**819.764**	**2137.616**	0.560	**16,884.989**	*5.940*	955.910	1575.630	0.562	15,601.694
	ISID-w/o	4.628	*863.925*	2190.761	0.552	*17,794.594*	**5.719**	*888.376*	1522.490	0.609	*14,499.446*

The results table is divided into two parts; the left half are the results on the Japan-Prefectures dataset, while the right half are the results on US-Regions. The runtime is determined by the total time of the entire TSCV process for each model; the other four measurements are calculated as the average values. The notion ↓ means lower the better and ↑ means higher the better. For ease of reading, the optimal and suboptimal values under each comparison are identified in bold and italic font, respectively.

In the comparison of running time, mainly for complex deep models are analyzed, traditional models are not compared in terms of speed due to the fast-computational process. In a total of six comparison experiments with two datasets and three prediction ahead steps, models ISID and ISID-w/o used in this study are alternately the fastest and second fastest of the deep models. Except on the prediction task at $\mathrm{\alpha }=10$ on Japan-Prefectures, LSTNet becomes the second fastest deep model, while ISID is still the fastest. In most scenarios, LSTNet is the third fastest deep model; the ISID and ISID-w/o models have an average speed-up of 16.4% compared to it, demonstrating the proposed method’s time efficiency.

For the three comparisons on Japan-Prefectures, it can be seen that ISID and ISID-w/o are either optimal or suboptimal on most of the measurements, except for the relatively better performance of Cola-GNN on PCC. On the other hand, the experimental results on US-Regions show that the light ISID-w/o is superior compared to ISID and outperforms most other models in terms of primary measurements on the tasks of $\mathrm{\alpha }=3$ and $5$. However, the performances of ISID and ISID-w/o are relatively worse at $\alpha =10$ on US-Regions, indicating that the ISID model is more suitable for short and medium-term prediction tasks, and the model effectiveness gradually declines as the prediction ahead timestep increases.

To further analyze the models’ performance in high utility short-term epidemic prediction scenarios such as $\alpha =3$, the two datasets are sequentially divided into training sets (60%), validation sets (20%), and test sets (20%), all models are retrained and compared using DM test. The DM test results can also be found in Appendix A2.

### SHAP analysis results

Since each input sample is a multivariate time-series epidemic data containing multiple regions and historical steps, not general two-dimensional tabular data, this study innovatively adopts the idea of image interpretability to visualize the model decision process for SHAP analysis. First, SHAP analysis was performed on the Japanese-Prefectures dataset for the prediction results of the last sample. The comparison is mainly made using the interpretable results of Cola-GNN and ISID with training epochs equal to $100$, and the prediction mode of each model is set to predict one future timestep ($\alpha =1$) using the past 20 steps ($\Delta =20$).

**Figure 2:**
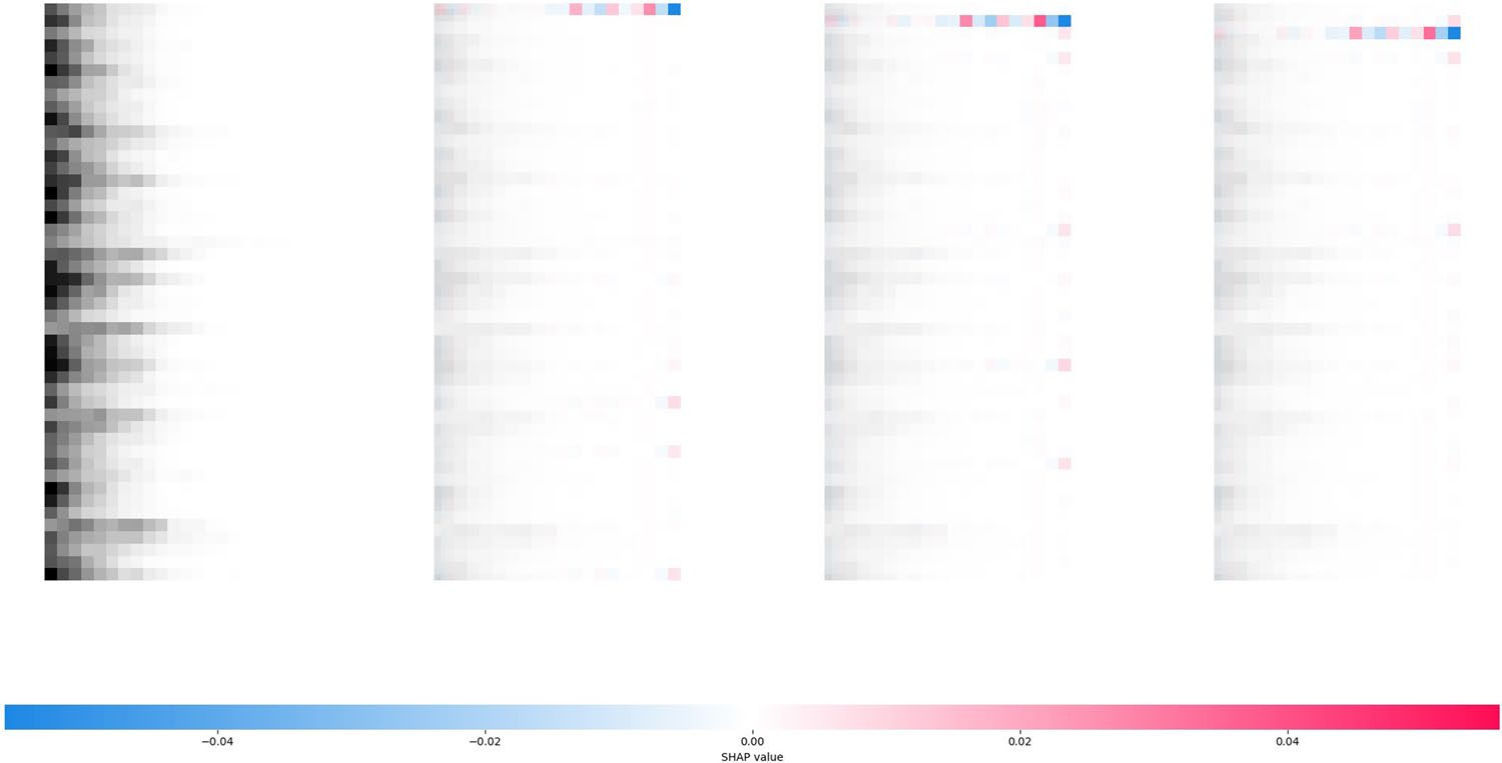
SHAP analysis for one prediction result of Cola-GNN on the Japanese-Prefectures.

The result of the Cola-GNN model is shown in Fig. 2. There are four subplots in the figure, with the actual data input on the leftmost side (epidemic data for the 47 regions for the 20 weeks prior to this forecast). Starting from the second subplot, which corresponds to the results of the SHAP analysis for three particular Japanese prefectures, the blue color in the figure means that the past values have a negative impact on the current forecast, and the red color means a positive impact. Each prefecture's historical data corresponds to the row of the leftmost subplot. For example, the first region corresponds to the first row, and the second region corresponds to the second row, and so on. For Cola-GNN, the essential basis for predicting one timestep is the historical epidemic data for the selected prefecture during past steps, i.e., in the range $\Delta \le 10$. The second important thing is the data of other regions for the most proximal moments of $\Delta =1$. The patterns analyzed by the SHAP result illustrate the prediction preferences of Cola-GNN.

**Figure 3:**
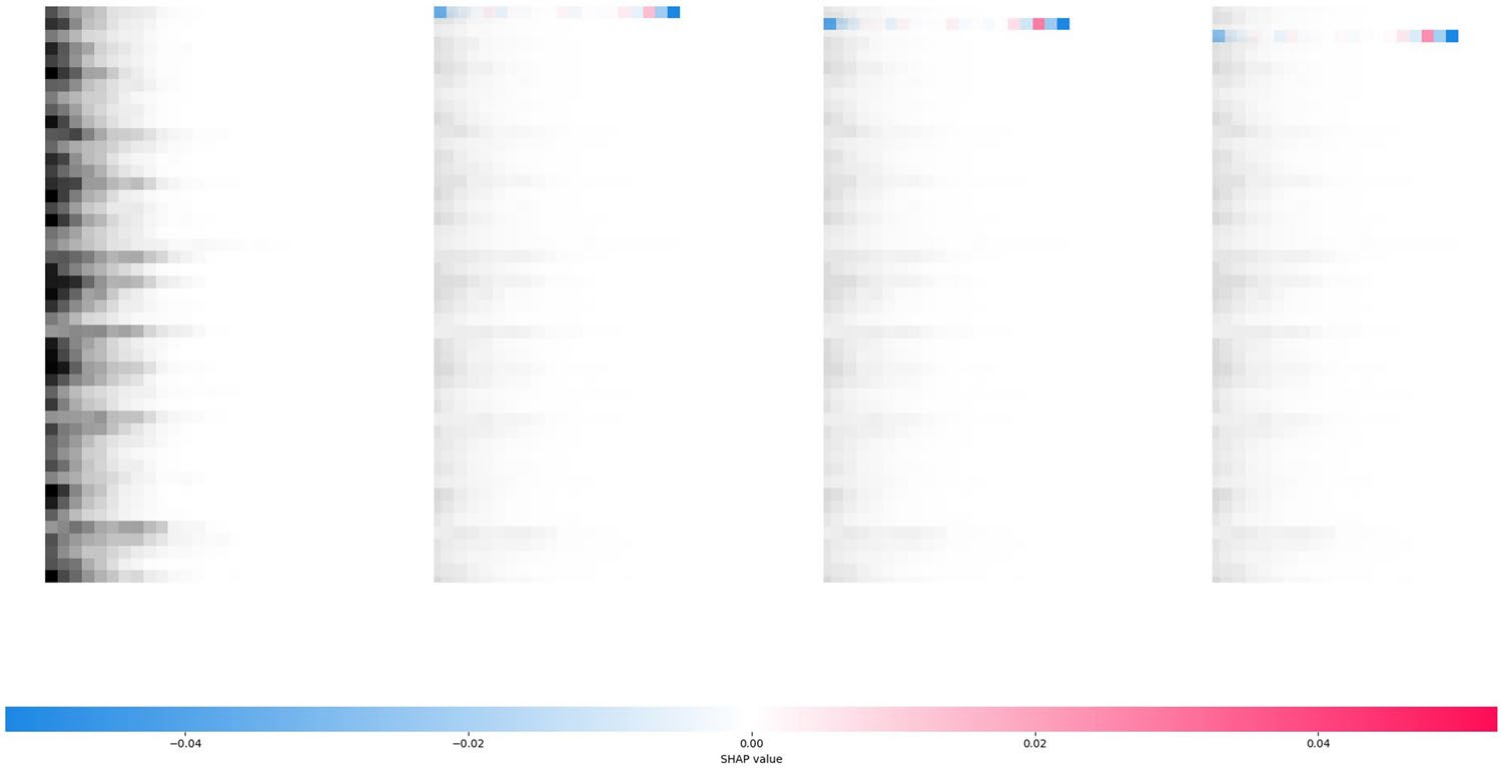
SHAP analysis for one prediction result of ISID on the Japanese-Prefectures.

The SHAP analysis result of ISID on the selected sample is shown in Fig. 3. The primary difference between ISID and Cola-GNN is that ISID in SHAP results does not focus only on the proximity step for a specific prefecture's historical data itself; on the contrary, the data of the distant step ($\Delta =20$) also has an important impact on the prediction results. Second, ISID does not explicitly use data from other regions to assist in the prediction of selected regions, as only the given region's historical epidemic values can be seen to have a SHAP impact on itself, with other regions showing insignificant gray color. However, this does not mean that ISID fails to learn the contagion of the epidemic between areas; on the contrary, the spatial transmission relationship is learned by the spatial identity matrix.

**Figure 4:**
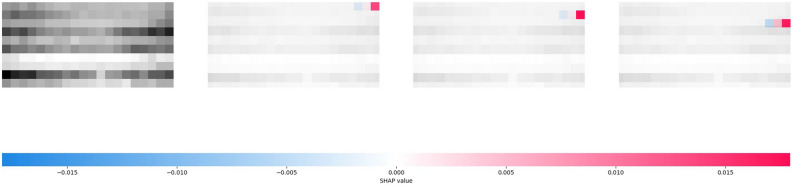
SHAP analysis for one prediction result of Cola-GNN on the US-Regions.

**Figure 5:**
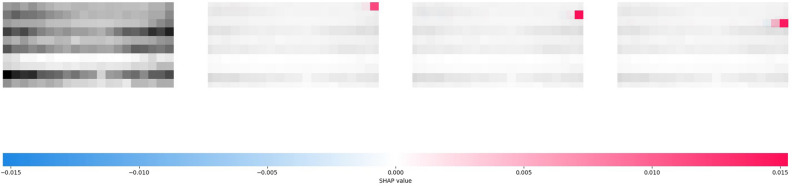
SHAP analysis for one prediction result of ISID on the US-Regions.

Further, the comparative results of SHAP analysis for ISID and Cola-GNN on the US-Regions dataset are shown in Figs. 4 and 5. In this case, the prediction of COLA-GNN is also mainly based on the recent historical data of the specific region itself, and the difference between the two models is not evident. However, in the visualization of the spatial identity matrix, it can be noticed that ISID still learns the proximity relationship between spaces, which can be found in Appendix A4.

### Discussion

The ISID model based on the simple fully connected neural network with spatial identity matrix proposed in this study achieves the effectiveness of Cola-GNN and even outperforms it in the short-term epidemic prediction task. Based on the analysis of post-hoc interpretable methods, it can be found that the decision logic of ISID is significantly different from graph representation learning approaches. This means such a question is highly valuable: a more complex or user-friendly model? The usual opinion would be that deep learning, while pursuing performance by deepening the number of neural network layers and increasing structural complexity, has the inevitable consequence of decreasing interpretability, with a regrettable tradeoff between predictive accuracy and model user understandability.

However, the results of ISID and its variant in this study illustrate that this unpleasant contradiction can be resolved more flexibly. According to the experimental results, ISID performs better and more robustly on the Japan-Prefectures dataset with more regions; this suggests that in the case of relatively complex spatial relationships, the spatial identity matrix can better learn the contagion process by discovering the clustering and sparsity characteristics between the spaces. It overcomes the inadequacy of the traditional GNNs in which expert experience and domain knowledge are used to construct fixed graph structures. The spatial identity matrix can perform state updates in a more dynamic and learnable manner, improving the spatial sensitivity of the model. The residual-connected multilayer perceptron further improves the model’s running speed and generalization ability.

In this regard, using the most basic fully connected neural network, leaving aside the complex and novel architecture, is nevertheless likely to achieve good results on relatively simple multivariate time series forecasting tasks, especially when combined with improvements such as historical sliding windows and residual structures. One of the most significant advantages of fully connected neural networks is that, ideally, they rely on the simplest structure yet may approximate complex nonlinear mapping relationships^41^. At the same time, traditional fully connected networks are also more easily understood by non-computer experts than deep learning structures such as GNNs. Further, with the help of the spatial identity matrix, the model’s interpretability can be improved. Spatial collinear proximity relations are transformed from the priori graph construction in graph representation learning into a trainable learning spatial identity matrix, bridging the possible shortcomings of expert experience and domain knowledge.

Further, the interpretable analysis results of ISID are also valuable for outbreak prevention and risk management in practice. From SHAP, it can be seen that ISID pays more attention to the future impact of the epidemic infection numbers at the most proximate time point versus several months ago, illustrating the cyclical and seasonal nature of the epidemic. Therefore, ISID forecasts can be used to estimate possible future infection peaks and consequently adjust production plans for anti-epidemic supplies, stockpile sufficient resources, and develop contingency measures before the arrival of possible high-risk seasons. As seen from the results of the T-SNE analysis, the epidemic contagion relationship in the ISID perspective is varied in different regions. Thus, it is possible to focus more on the closest regions in the T-SNE representation when epidemics break out in a specific region, rather than just the regions that are nearer in reality. This will enable better use of interpretable results to manage epidemic risk and to deliver urgent protective materials to areas in greater need.

Nevertheless, the ISID proposed in this study still has some limitations. First, the results of long-term forecasting are still not good enough, and the accuracy needs to be further improved in the future while cautiously building the model, especially to control the increase of model complexity. Second, model distillation techniques^42,43^ can be considered in the future to improve the generalization ability of the model while streamlining the model structure, making it applicable to more epidemic prediction scenarios.

## Conclusion

This paper proposed a novel interpretable epidemic prediction model ISID, which is constructed based on fully connected neural networks and spatial identity matrix for predicting the number of epidemic infections that vary dynamically in time and space. Unlike deep graph neural network models, which might be overly complex for public health experts, ISID only employs a simple network architecture while achieving efficiency and performance similar to GCNs. In particular, considering the contagion of epidemics across time–space, ISID utilizes a learnable identity matrix of spatial relationships that allows for better mining of potential spatio-temporal correlations between regions. Moreover, ISID has post-hoc interpretability to identify the crucial indicators on prediction from multivariate time series inputs of infection numbers. The effectiveness of the ISID model is demonstrated by comparison experiments on two epidemic-related datasets from the United States and Japan.

In the future study, there are two main concerns. First, this study mainly focuses on influenza regression prediction, but the risk components included in epidemics are multitudinous beyond infection numbers. Especially in the perspective of classification tasks: outbreak level, severity, priority risk areas, scale, and other prediction goals are probabilistic and under uncertainty. Therefore, extending the proposed ISID model in the context of multi-classification or multi-label prediction is necessary by modifying the loss function, output mapping layer, etc., to make ISID further adaptable to a broader range of epidemic risk analysis tasks. Second, more external drivers impacting the epidemic, such as population movement, social factors, economic factors, and policy instruments, will be considered. In addition, how to propose more operational epidemic intervention strategies to contain and slow down the predicted growth spike in the number of infections is also a critical post-prediction issue.

### Supplementary Information

Supplementary Information.

## Data Availability

All the data are available upon reasonable request by contacting the corresponding author.
