# Supplementary material for: Interpretable spatial identity neural network-based epidemic prediction
Source: Sci Rep. 2023 Oct 24;13:18159. doi: 10.1038/s41598-023-45177-1 (PMC10598274; doi:10.1038/s41598-023-45177-1)
Supplement: Supplementary file 1 — Supplementary Information. [file 41598_2023_45177_MOESM1_ESM.docx]

# Supplementary Appendix

## A.1 SHapley Additive exPlanations method

The SHapley Additive exPlanations (SHAP) method used in this study has been widely adopted in the interpretable machine learning field^1^. SHAP is designed to explain the predictions of machine learning models at both the local and global levels based on game theory. SHAP can calculate the optimal Shapley values and explain the prediction of any instance by analyzing the contribution of each feature, which can be represented as Eq.A1：

| $g\left( z^{'} \right)=\phi_{0}+\sum_{j=1}^{M} \phi_{j}z_{j}^{'}$, | (A1) |
| --- | --- |

where $g$ denotes the explanation model, $z^{'}\in\left\{ 0,1 \right\}^{M}$ is the coalition vector, $M$ is the maximum coalition size, and $\phi_{j}\mathbb{\in R}$ is the feature imputed Shapley value of feature $j$.

## A.2 Diebold Mariano test

In addition to the general error assessment approaches, many machine learning-based time series prediction studies adopt the Diebold Mariano (DM) test to evaluate the model reliability^2^. Specifically, the DM test is mainly applied to the pairwise comparison of two models' prediction ability when the ground truth is known, the comparative results are given by the DM statistic. The calculation can be represented as Eq. A2 to A5:

| $d_{i}=\left\vert\hat{y}_{1i}-y_{i} \right\vert-\left\vert\hat{y}_{2i}-y_{i} \right\vert$ | (A2) |
| --- | --- |
| $\bar{d}=\frac{1}{n}\sum_{i=1}^{n} d_{i}$ | (A3) |
| $\gamma_{k}=\frac{1}{n}\sum_{i=k+1}^{n} (d_{i}-\bar{d})(d_{i-k}-\bar{d})$ | (A4) |
| $\mathrm{DM}\boldsymbol{=}\frac{\bar{\boldsymbol{d}}}{\sqrt{{\boldsymbol{(}\boldsymbol{\gamma}_{\boldsymbol{0}}\boldsymbol{+2}\sum_{\boldsymbol{k=1}}^{\alpha\boldsymbol{-1}} \gamma_{k}\boldsymbol{)}}/\boldsymbol{n}}}$ | (A5) |

where $y_{i}$ denotes the $i$-th ground truth in the test set, $\hat{y}_{1i}$ and $\hat{y}_{2i}$denote the prediction from model $1$ and model $2$. Specifically, $d_{i}$ means the forecasting error differential between two models and $\bar{d}$ represents the mean of series ${\{d_{i}\}}_{i=1}^{n}$. The autocovariance at lag $k$ can be calculated based on the autocorrelation function $\gamma_{k}$. Finally, the DM statistic is computed from $n$, $\bar{d}$, $\gamma_{k}$, and prediction ahead timestep $\alpha$. Once the cumulative sum of prediction errors for model $1$ is greater than model $2$, then the $\bar{d}$ exceeds $0$. Under the same test set, the larger the DM statistic of the two models, the greater the difference in model effects.

The DM test results of all models on two datasets are shown in Fig.A1 and A2. Since DM performs paired tests, the results are represented as a matrix heatmap, where each cell $\mathrm{DM}_{\mathrm{ij}}$ implies the result of comparing the $i$-th model with the $j$-th, and if $\mathrm{DM}_{\mathrm{ij}}<0$, it means that the error of model $i$ is smaller compared to $j$ according to Eq. A2 to A5. It can be seen that ISID and ISID-w/o perform relatively best for $\alpha= 3$.


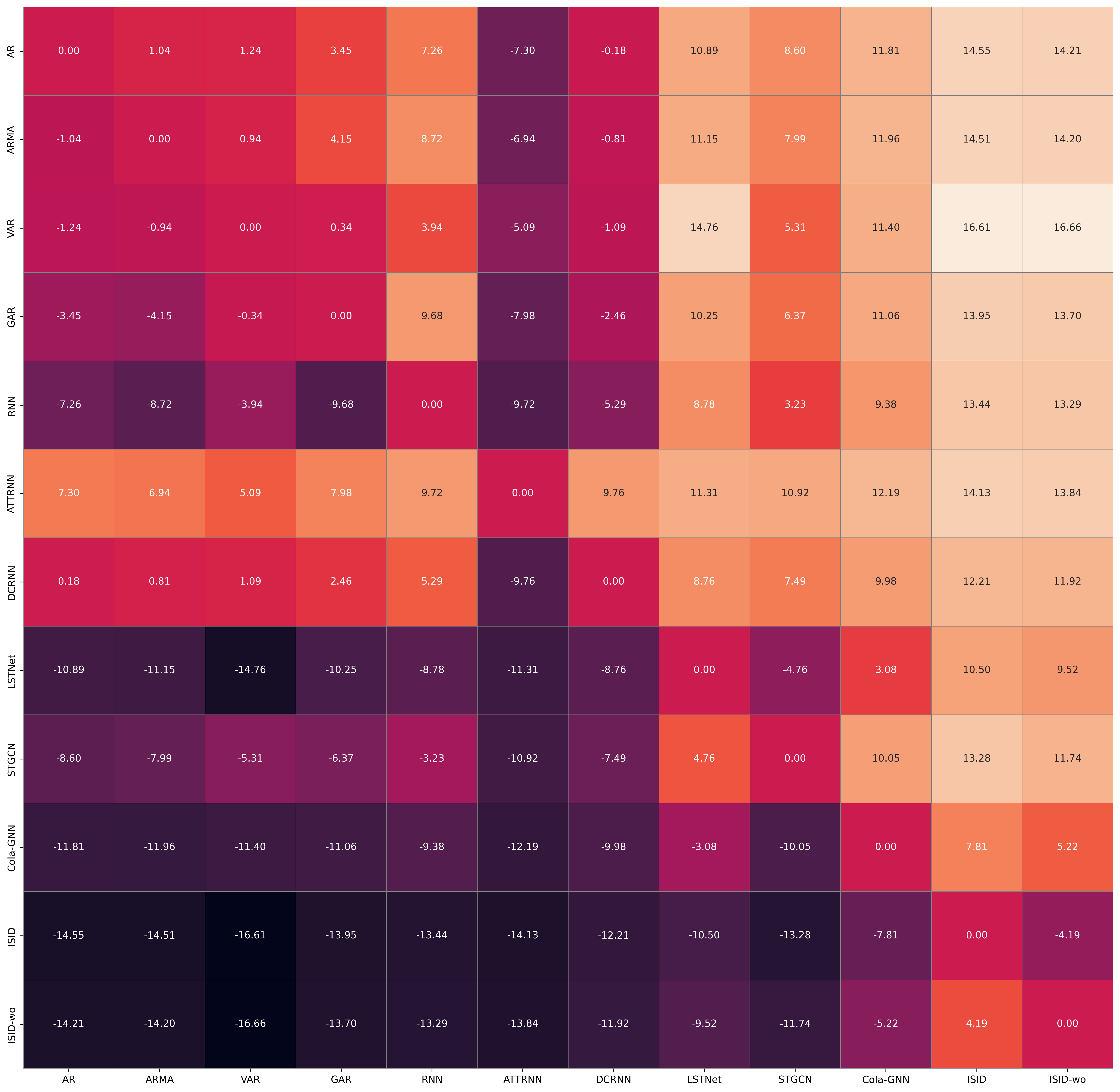


Figure A1: DM-test between models on the Japanese-Prefectures dataset ($\alpha=3$)


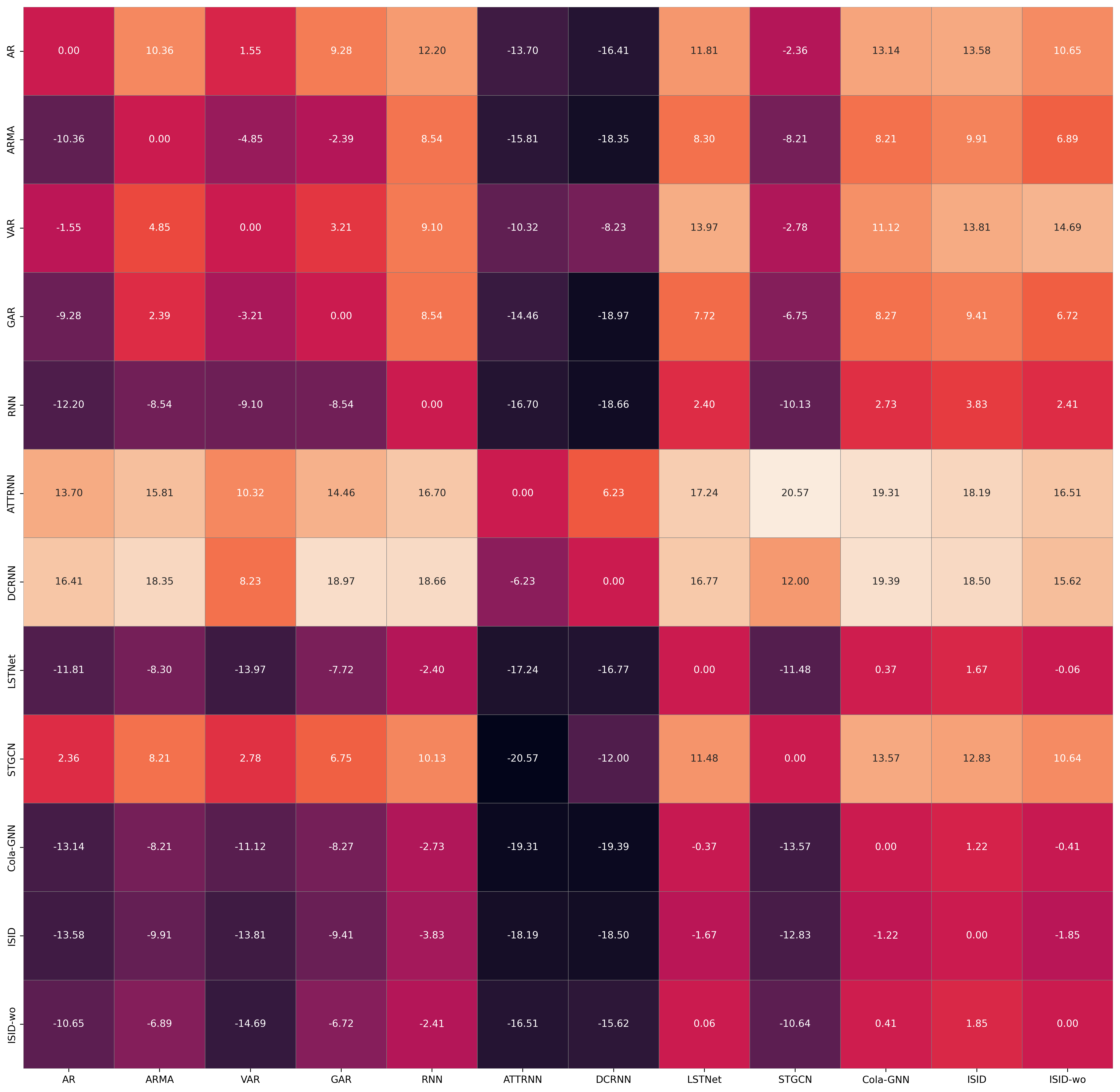


Figure A2: DM-test between models on the US-Regions dataset ($\alpha=3$)

## A.3 Time series cross-validation method

Time Series Cross-Validation (TSCV) is the most used method to test the model robustness for prediction tasks with chronological constraints. TSCV divides time series data into multiple slices in temporal order, and for each training-validation-test process, the previous $k$ slices are used as training, and the $k+1$ slice is used as the validation set. The detailed TSCV method used in this study is shown in Fig.A3.


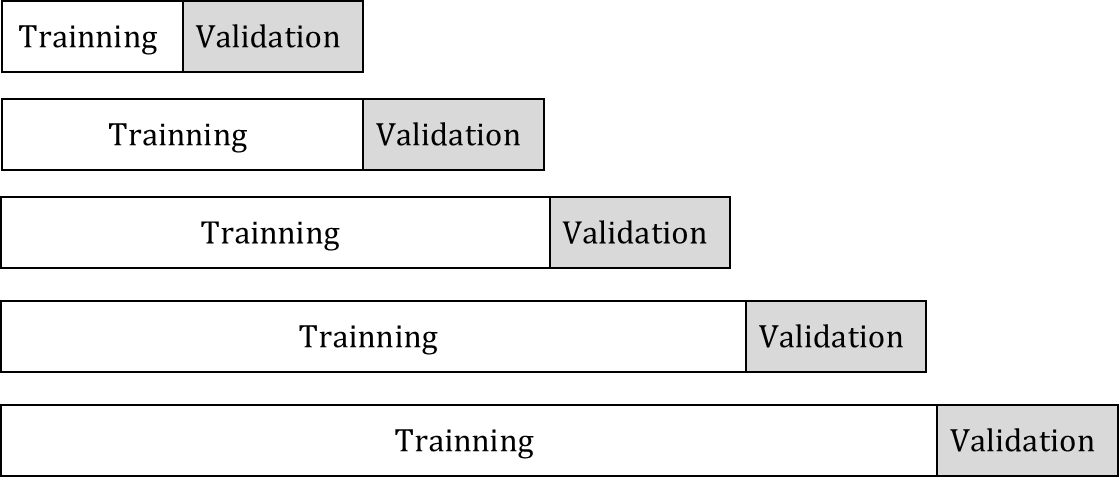


Figure A3 Representation of the TSCV (splits=5) implemented in this study

A new supervised learning process begins in each cross-validation process, and the recent validation split is included to form a new training set. It is worth noting that to ensure the experiment's fairness, the test set is fixed in this study, and each model's performance is reported as the average across all TSCV subsets.

## A.4 Visualization of ISID's spatial identity matrix through T-SNE

The trained spatial identity matrixes of ISID on two datasets with $\alpha=1$ are visualized in Fig.A4 and A5, based on the t-distributed Stochastic Neighbor Embedding (T-SNE) method ^3^.


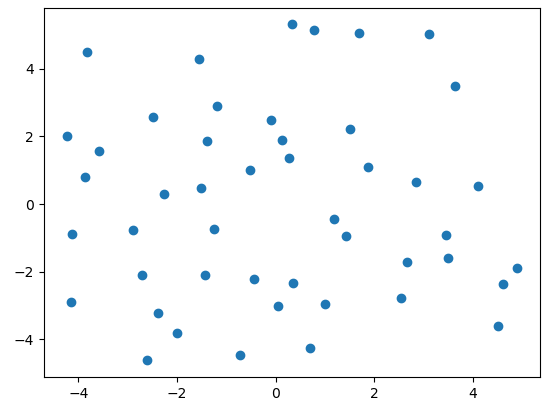


Figure A4: Visualization of ISID's spatial identity matrix based on T-SNE (Japanese-Prefectures). The horizontal and vertical axes record the ranges of prefectures' spatial identities after dimensionality reduction.

It can be seen that the ISID model learns the proximity relationship for different regions. In the reduced-dimensional planar representation, each prefecture corresponds to a point, and the closer the points are, the more likely they are to interact with each other, and the epidemic is more likely to be contagious between them. Thus, the ISID model uses a trainable spatial identity matrix for learning the spatio-temporal contagion of epidemics, which achieves similar results to more complex deep learning models. According to Figure A5 for the T-SNE analysis of the spatial identity matrix in ISID, it can be found that the ISID model still learns the difference among regions, some of which are closer to the clusters, while some are relatively more distant. It can be considered that ISID gives more prediction interpretation compared to Cola-GNN.


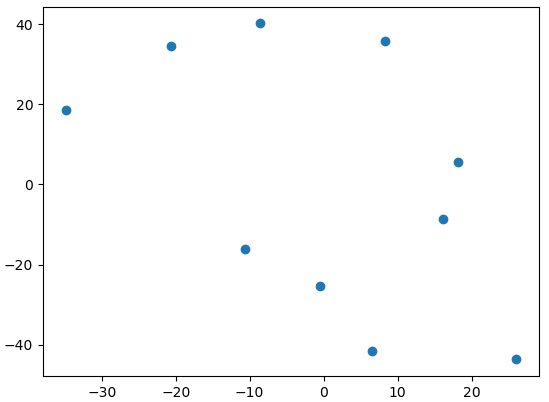


Figure A5: Visualization of the ISID's spatial identity matrix based on T-SNE (US-Regions)

## Reference

1. A game theoretic approach to explain the output of any machine learning model. https://github.com/shap/shap.

2. Chen, H., Wan, Q. & Wang, Y. Refined Diebold-Mariano Test Methods for the Evaluation of Wind Power Forecasting Models. *Energies* **7**, 4185–4198 (2014).

3. Van der Maaten, L. & Hinton, G. Visualizing data using t-SNE. *Journal of machine learning research* **9**, 2579–2605 (2008).
